# Overexpression of P4HA1 Is Correlated with Poor Survival and Immune Infiltrates in Lung Adenocarcinoma

**DOI:** 10.1155/2020/8024138

**Published:** 2020-11-24

**Authors:** Haiting Zhou, Yi He, Lingling Li, Cheng Wu, Guoqing Hu

**Affiliations:** ^1^Department of Oncology, Tongji Hospital, Tongji Medical College, Huazhong University of Science and Technology, Wuhan, Hubei 430030, China; ^2^Department of Orthopedics, Tongji Hospital, Tongji Medical College, Huazhong University of Science and Technology, Wuhan, Hubei 430030, China

## Abstract

Lung adenocarcinoma (LUAD) is a major pathological type of lung cancer. Understanding the mechanism of LUAD at the molecular level is important for a clinical decision. In this study, we use bioinformatic analysis to explore the prognostic value of P4HA1 in lung adenocarcinoma (LUAD) and the relationship with prognosis and tumor-infiltrating immune cells (TIICs). The results showed that the expression of P4HA1 was significantly higher in tumor tissues than in normal tissues for LUAD patients. Upregulated P4HA1 was related to stage and T classification. Kaplan-Meier analysis indicated that upregulation of P4HA1 was significantly related to worse overall survival (OS). Univariate and multivariate Cox analysis indicated P4HA1 remained to be an independent prognostic factor. GSEA showed that several cancer-related and immune-related signaling pathways exhibited prominently differential enrichment in P4HA1-high expression phenotype. In addition, the expression of P4HA1 was significantly correlated with proportion of several TIICs, particularly B cells and CD4+ T cells. In conclusion, our study confirmed that P4HA1 is a promising biomarker of poor prognosis and relates to immune infiltrates in LUAD.

## 1. Introduction

Lung cancer is the main cause of cancer-associated mortality and afflicting the global population [1]. Based on histological type, lung cancer is classified as non-small cell lung cancer (NSCLC) and small cell lung cancer (SCLC). NSCLC occupies 85% of lung cancer cases, which is divided into lung adenocarcinoma (LAUD), lung squamous cell carcinoma (LUSC), and large cell lung carcinoma [2]. In recent years, there has been a clear upward trend in LUAD, and LUAD has substituted LUSC as the most common histological subtype [3]. Although breakthroughs have been gained in diagnoses and treatments, the 5-year survival rates for LAUD are still low, and the recurrence rate remains dissatisfactory [4]. Most patients are at the advanced stages when first diagnosed and lose the opportunities of surgery. In the past decades, cytotoxic chemotherapy was the primary therapy for advanced patients, but the efficacy reached a plateau. Although the treatment of targeting driver mutations involving epidermal growth factor receptor (EGFR) and anaplastic lymphoma kinase (ALK) fusion oncogenes showed significant survival benefits, limited patients could benefit from them [5, 6]. Recently, several immune checkpoint blockers including programmed death 1 (PD-1) and its ligand PD-L1 or cytotoxic T-lymphocyte antigen-4 (CTLA-4) exhibit extraordinary clinical effects in NSCLC [7, 8]. Tumor-infiltrating immune cells (TIICs) have been a hot area of research along with the rapid development of immunotherapy [9, 10]. Increasing evidence proved that TIICs play pivotal roles in the development and progression of several human cancers [11]. However, the specific mechanism of TIICs in lung adenocarcinoma remains unclear.

Collagen constitutes and affects the tumor microenvironment (TME) via remodeling extracellular matrix (ECM) by degradation and redeposition. Moreover, collagen facilitates malignancy infiltration, invasion, migration, and angiogenesis [12]. Prolyl hydroxylation influences the structure and function of proteins by posttranslational protein modification. Collagen constitutes the primary substrate of prolyl hydroxylation [13]. Prolyl-4-hydroxylase (P4H) is of great significance for collagen biosynthesis, because 4-hydroxyproline residues are essential for the stability of the collagen triple helix [14]. P4Hs are tetrameric isoenzymes comprising two alpha subunits (catalytic) and two beta subunits (encoded by P4HB). The P4H family contains P4HA1, P4HA2, P4HA3, and P4HB, and all of these genes enable encoding proteins in collagen biosynthesis [13]. It is worth noting that P4HA1 is the primary isoform in most cells and determines the activities of P4H [15]. Previous evidence indicated that elevated P4HA1 expression was related to poor prognosis in some solid cancers, such as pancreatic cancer, head and neck cancer, high-grade gliomas, breast cancer, prostate cancer, and oral cancer [16–20]. However, the correlation between P4HA1 expression and LUAD remains uninvestigated. So, this study was aimed at shedding light on the prognostic value and underlying mechanism of P4HA1 in LUAD. Besides, for the first time, we investigated the association between P4HA1 expression and abundance of TIICs.

In this study, we demonstrated that P4HA1 was upregulated in tumor tissues compared to normal tissues in LUAD. Overexpression of P4HA1 was related to adverse prognosis. GSEA indicated that the P4HA1-high expression phenotype correlated with several cancer-related and immune-related signaling pathways, such as ubiquitin-mediated proteolysis, P53 signaling pathway, mismatch repair, nucleotide excision repair, cell cycle, DNA replication, TGF-*β* signaling pathway, PI3K/AKT/mTOR signaling pathway, IL1 mediated signaling pathway, regulation of macroautophagy, targets in activated B lymphocyte, and hypoxia. Analysis of immune infiltration showed that expression of P4HA1 was related to infiltrating levels of several TIICs, particularly B cells and CD4+ T cells.

## 2. Materials and Methods

### 2.1. Data Collection and Preprocessing

The level 3 gene expression data (project: TCGA-LUAD, workflow type: HTSeq-FPKM) and corresponding clinical information were downloaded from The Cancer Genome Atlas (TCGA) database, which contained 535 tumor samples and 59 normal samples. Then, FPKM data was converted into TPM data (log2-transformed) for the following analysis. Four microarray datasets (GSE30219, GSE31210, GSE68465, and GSE72094) from the Gene Expression Omnibus (GEO) database (https://www.ncbi.nlm.nih.gov/geo/) were reprocessed and normalized using Limma package in R and were used to validate the results.

### 2.2. P4HA1 Gene Expression Analysis

The expression of P4HA1 in various cancers, including LUAD, was analyzed using the TIMER (https://cistrome. shinyapps.io/timer/) database with TCGA data. The TCGA-LUAD dataset, including 535 tumor tissues and 59 normal tissues, was applied for P4HA1 differential expression analysis. GSE30219, including 85 normal tissues and 14 normal tissues, was used for verification.

### 2.3. Profiling of Genes Coexpressed with P4HA1

LinkedOmics (http://www.linkedomics. org/login.php) is a public database for online analysis of multiomics data across 32 cancer types from the TCGA database [21]. Coexpressed genes with P4HA1 were identified using Pearson's correlation analysis. The association results were displayed in volcano plot and heat maps. We further validated the results using Pearson's correlation analysis and Spearman correlation analysis in the cBioPortal database (http://cbioportal.org). A protein-protein interaction (PPI) network was constructed based on the Pearson correlation coefficient (∣cor | >0.4, *P* < 0.01) between P4HA1 and the coexpressed genes to predict the potential targets of P4HA1in the STRING database (https://string-db.org/)[22]. In addition, we used Cytoscape (v3.8.0) to visualize the network.

### 2.4. The Relationship between P4HA1 Expression and Prognosis in LUAD

The clinical information of LUAD was downloaded from the TCGA database, which contained the gender, age, TNM stage, follow-up time, and survival status of 513 patients. Then, we explored the effect of aberrant expression of P4HA1 on clinicopathologic characteristics. Patients with complete clinical information and follow-up time > 30 days were used further for survival analysis. Three datasets (GSE31210, GSE68465, and GSE72094) with follow-up information were used for validation.

### 2.5. Gene Set Enrichment Analysis (GSEA)

GSEA was performed using GSEA software (v.4.0.3), which is a powerful method to test whether a set of previous defined genes shows statistical significance between two biological states [23]. In this study, GSEA was carried out to explore different function phenotypes between high-expression and low-expression P4HA1 groups. We chose c2.cp.kegg.v6.0.symbols.gmt, c5.go.v7.2.symbols.gmt and h.all.v7.2.symbols.gmt as the annotated gene set. FPKM was converted into TPM data (log2 transformed) for GSEA. We set 1000 times for gene set permutations per analysis. Evaluation of enrichment pathways for each phenotype used nominal *P* value, false discovery rate (FDR) *q* values, and normalized enrichment scores (NES).

### 2.6. Evaluation of Tumor-Infiltrating Immune Cells

TIMER was applied to evaluate the abundance of TIICs. We used the “gene module” to investigate the relationship between P4HA1 expression and specific TIIC subsets in LUAD, which was displayed by scatterplots with Spearman's correlation coefficients. In order to visualize the survival difference between the expression levels of P4HA1 in each immune subset, we utilized the “survival module” to draw survival curves for immune infiltrating cells and P4HA1 with log-rank test. “SCNA module” was applied for investigating the relationship between somatic copy number alterations (SCNAs) of P4HA1 and TIICs in LUAD. SCANs were analyzed based on GISTIC 2.0, containing arm-level deletion, deep deletion, high amplification, diploid/normal, and arm-level gain. The distribution of each immune subset at different somatic copy number status of P4HA1 in LUAD was visualized by box plots. Two-sided Wilcoxon rank-sum test was used to estimate the statistical significance.

Then, we used the TISIDB (http://cis.hku.hk/TISIDB/index.php) database [24] to further explore the relations between the P4HA1 expression with immune subtypes and proportions of 28 TIICs. Spearman's test was utilized to evaluate associations between P4HA1 expression and proportion of TIICs. *P* value < 0.05 was considered statistically significant.

### 2.7. Statistical Analysis

All statistical analyses were processed using R software (v.3.6.1). All data was preprocessed by Perl. First, we made use of Mann-Whitney *U* test and Wilcoxon signed-rank test to probe the differential expression of P4HA1 between matched and unmatched samples. Next, we utilized Mann-Whitney *U* test, Kruskal-Wallis test, and logistic regression to analyze the relationship between clinicopathological characteristics and the P4HA1 expression. The association between P4HA1 expression and overall survival was analyzed by Kaplan-Meier method using the log-rank test. Univariate cox analysis was used to evaluate the correlation between overall survival and clinicopathological characteristics. Multivariate cox analysis was used to further evaluate the impact of P4HA1 expression on the prognosis independent of other clinicopathological features (such as age, gender, stage, tumor status, lymph node status, and distant metastasis status). The cut-off value of P4HA1 expression depended on the median value. *P* < 0.05 was considered statistically significant.

## 3. Results

### 3.1. P4HA1 Expression at mRNA Level in Various Cancers

**Figure 1:**
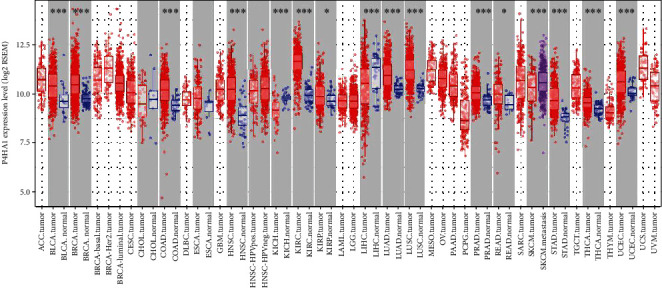
P4HA1 expression levels in different types of human cancers. The P4HA1 expression was analyzed in various tumor tissues and adjacent normal tissues through the TIMER database. (^∗^*P* < 0.05, ^∗∗^*P* < 0.01, and ^∗∗∗^*P* < 0.001).

As shown in Figure 1, P4HA1 was significantly upregulated in many solid malignant tumors, such as BLCA (bladder urothelial carcinoma), BRCA (breast invasive carcinoma), COAD (colon adenocarcinoma), HNSC (head and neck squamous cell carcinoma), KIRC (kidney renal clear cell carcinoma), KIRP (kidney renal papillary cell carcinoma), LUAD (lung adenocarcinoma), LUSC (lung squamous cell carcinoma), PRAD (prostate adenocarcinoma), READ (rectum adenocarcinoma), STAD (stomach adenocarcinoma), THCA (thyroid carcinoma), and UCEC (uterine corpus endometrial carcinoma). Conversely, P4HA1 was significantly downregulated in KICH (kidney chromophobe) and LIHC (liver hepatocellular carcinoma). The results indicated that P4HA1 might play different roles in a variety of tumors.

### 3.2. P4HA1 Expression in LUAD Tissues and Normal Tissues

**Figure 2:**
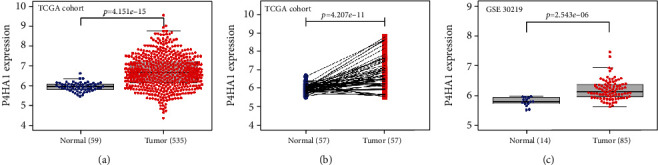
Expression level of P4HA1 in tumor and normal tissues. (a) Higher expression of P4HA1 was observed in tumor tissues than normal tissues in TCGA samples (*P* < 0.001). (b) Overexpression of P4HA1 was observed in 57 matched tumors than adjacent normal tissues in TCGA samples (*P* < 0.001). (c) Higher expression of P4HA1 was observed in tumor tissues than normal tissues in GSE30219 (*P* < 0.001).

We collected 535 tumor tissues and 59 normal tissues with gene expression data from the TCGA database. Then, we used Mann-Whitney *U* test to compare the differential expression between the normal and tumor groups. Significantly higher P4HA1 expression was found in tumor tissues compared with normal tissues (*P* < 0.001) (Figure 2(a)). We used the Wilcoxon signed-rank test to probe the expression of P4HA1 in 57 paired tumor and adjacent normal tissues. Overexpression of P4HA1 was also observed in tumor tissues (*P* < 0.001) (Figure 2(b)). The similar results were also found in a GEO dataset (GSE30219) (*P* < 0.001) (Figure 2(c)).

### 3.3. Identification P4HA1-Related Genes and PPI Network Construction

**Figure 3:**
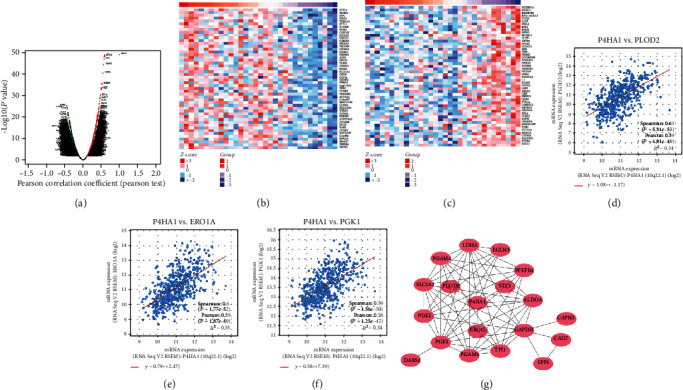
Coexpression genes of P4HA1 in LUAD. (a) Volcano plot showing the global P4HA1 highly correlated genes identified by Pearson test. Red indicates positively correlated genes, and green indicates negatively correlated genes. (b, c) Heat maps showing top 50 genes positively and negatively correlated with P4HA1. (d–f) The top 3 genes coexpressed with P4HA1 were verified in the cBioPortal database. (g) PPI network of P4HA1 was constructed using Cytoscape.

To better understand the biological significance of P4HA1 in LUAD, we used the function module of LinkedOmics to explore the coexpressed genes related to P4HA1 from 515 LUAD samples in TCGA. As shown in Figure 3(a), 3279 genes (dark red dots) were significantly positively correlated with P4HA1, while 4473 genes (dark green dots) were significantly negatively related with P4HA1 [false discovery rate (FDR) < 0.01]. The top 50 significant genes positively/negatively correlated with P4HA1 are shown in the heat maps (Figures 3(b) and 3(c)). Then, we used the coexpression module of the cBioPortal database to validate the result (Figures 3(d)–3(f)), which showed that PLOD2, ERO1A, and PGK1were highly relevant to P4HA1. Considering insight into the interaction between P4HA1 protein and effector proteins, we utilized the STRING online database to construct a PPI network based on the Pearson correlation coefficient (∣cor | >0.40, FDR < 0.01). The result was visualized by Cytoscape (Figure 3(g)).

### 3.4. Associations between P4HA1 Expression and Clinicopathological Characteristics in LUAD Patients

**Figure 4:**
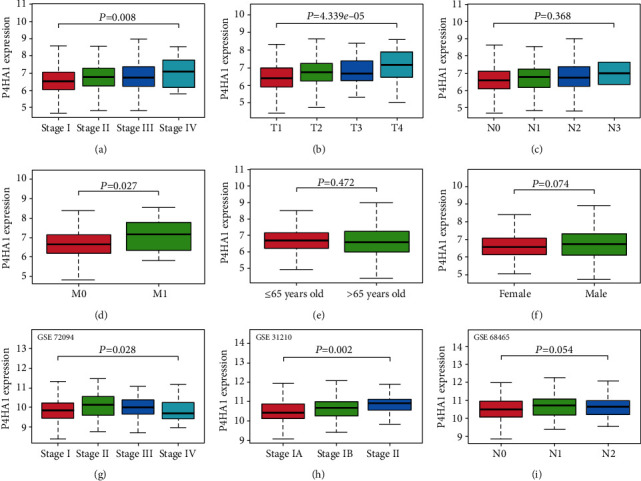
The relationship between P4HA1 expression and clinicopathologic features: (a) stage, (b) T classification, (c) N classification, (d) M classification, (e) age, and (f) gender in TCGA-LUAD cohort; (g) stage in GSE72094, (h) stage in GSE31210, and (i) N classification in GSE68465.

513 LUAD cases with clinical information from TCGA were analyzed. Elevated P4HA1 expression in LUAD was significantly associated with the stage (*P* = 0.008), T classification (*P* < 0.001), and M classification (*P* = 0.027) (Figures 4(a)–4(f)). Logistic regression used P4HA1 expression as an independent classified variable (based on the median value). The results demonstrated that overexpression of P4HA1 was prominently correlated with poor prognostic factors involving more advanced stage (stage I vs. II/III/IV, *P* = 0.009) and greater primary tumor size (T1 vs. T2/T3/T4, *P* < 0.001) and weakly related to metastasis status (M0 vs. M1, *P* = 0.066) (Table 1). Then, we verified the results in GSE72094, GSE31210, and GSE68465, as shown in Figures 4(g)–4(i). These results revealed that upregulated P4HA1 tend toward poor prognosis for LUAD patients.

### 3.5. Role of P4HA1 in LUAD Patient Survival

**Figure 5:**
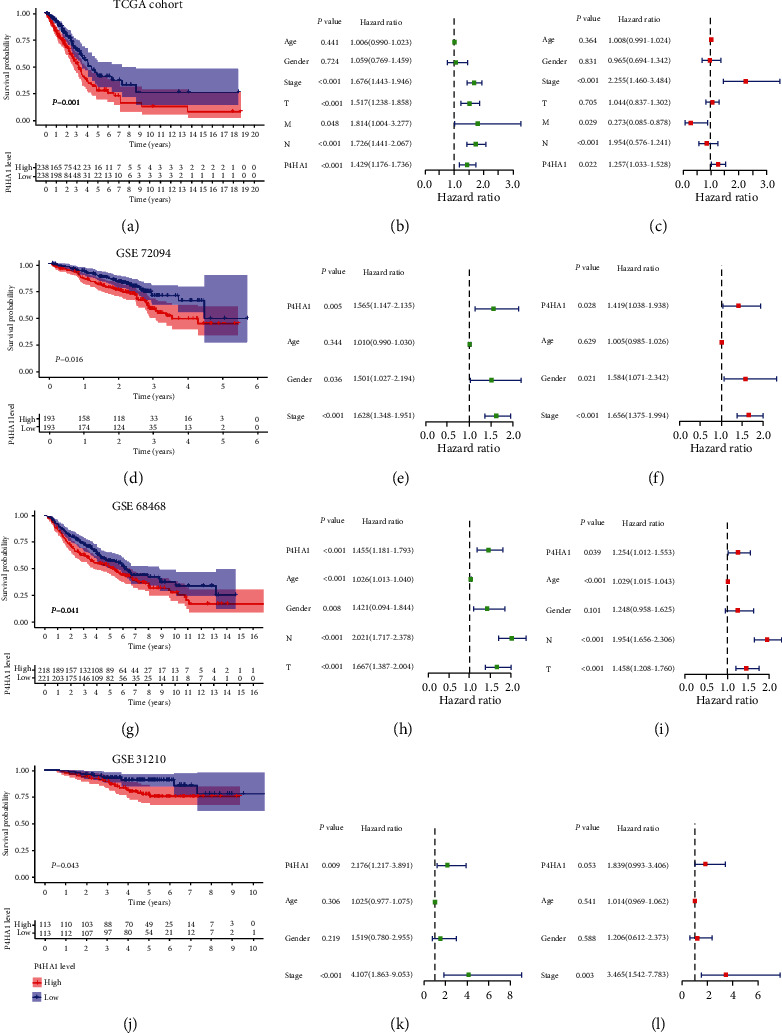
Survival-associated analysis of P4HA1 in LUAD. Overexpressed P4HA1 is correlated with poor prognosis and serves as an independent unfavorable prognostic factor in TCGA-LUAD cohort (a–c), GSE72049 cohort (d–f), GSE68468 cohort (g–i), and GSE31210 cohort (j–l). Green forest maps are univariate Cox analysis, and red forest maps are multivariate Cox analysis.

In the TCGA-LUAD cohort, Kaplan-Meier analysis showed that patients with high P4HA1 expression exhibited worse outcome than those with low P4HA1 expression (log-rank *P* = 0.001) (Figure 5(a)). The univariate Cox regression analysis uncovered that P4HA1 expression (HR = 1.43, 95% CI: 1.18-1.74, *P* < 0.001), stage (HR = 1.68, 95% CI:1.44-1.95, *P* < 0.001), T classification (HR = 1.52, 95% CI:1.24-1.86, *P* < 0.001), N classification (HR = 1.73, 95% CI: 1.44-2.07, *P* < 0.001), and M classification (HR = 1.81, 95% CI: 1.00-3.28, *P* = 0.048) were related to overall survival. We further established multivariate Cox regression model. The results indicated that P4HA1 expression (HR = 1.26, 95% CI: 1.03-1.53, *P* = 0.021), stage (HR = 2.25, 95% CI:1.46-3.48, *P* < 0.001), and M classification (HR = 0.27, 95% CI:0.09-0.88, *P* = 0.029) were independent predictive factors of prognosis for LUAD patients (Figures 5(b) and 5(c)). Then, we used the GSE72049 cohort (Figures 5(d)–5(f)), GSE68468 cohort (Figures 5(g)–5(i)), and GSE31210 cohort (Figures 5(j)–5(l)) to confirm the prognostic value of P4HA1. The results also indicated that overexpressed P4HA1 related to unfavorable outcome and served as an independent prognostic biomarker.

### 3.6. GSEA

**Figure 6:**
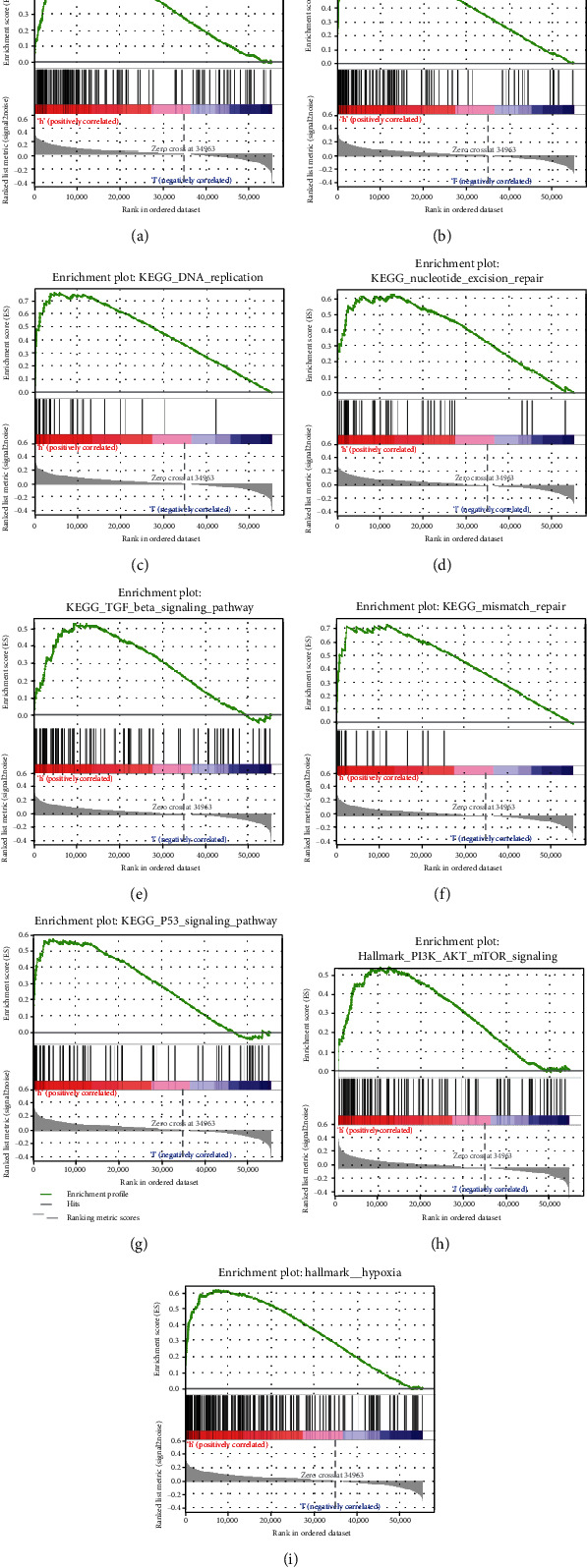
Enrichment plots from gene set enrichment analysis (GSEA). GSEA results showing ubiquitin-mediated proteolysis (a), cell cycle (b), DNA replication (c), nucleotide excision repair (d), TGF beta signaling pathway (e), mismatch repair (f), P53 signaling pathway (g), PI3K/AKT/mTOR signaling (h), and hypoxia (i) were differentially enriched in high P4HA1 expression phenotype.

In order to explore the signaling pathways that P4HA1 may regulate or influence, we performed GSEA between the high/low-P4HA1 expression groups based on TCGA data. GSEA revealed that significant differences caused by P4HA1 in multiple cancer-associated and immune-associated signaling pathways, and the details are showed in Table 2. Gene sets including ubiquitin-mediated proteolysis, P53 signaling pathway, mismatch repair, nucleotide excision repair, cell cycle, DNA replication, TGF beta signaling pathway, PI3K/AKT/mTOR signaling, and hypoxia were differentially activated in the P4HA1-high expression phenotype (Figures 6(a)–6(i)).

NES: normalized enrichment score; NOM: nominal; FDR, false discovery rate.

### 3.7. Association between P4HA1 Expression and Tumor-Infiltrating Immune Cells

**Figure 7:**
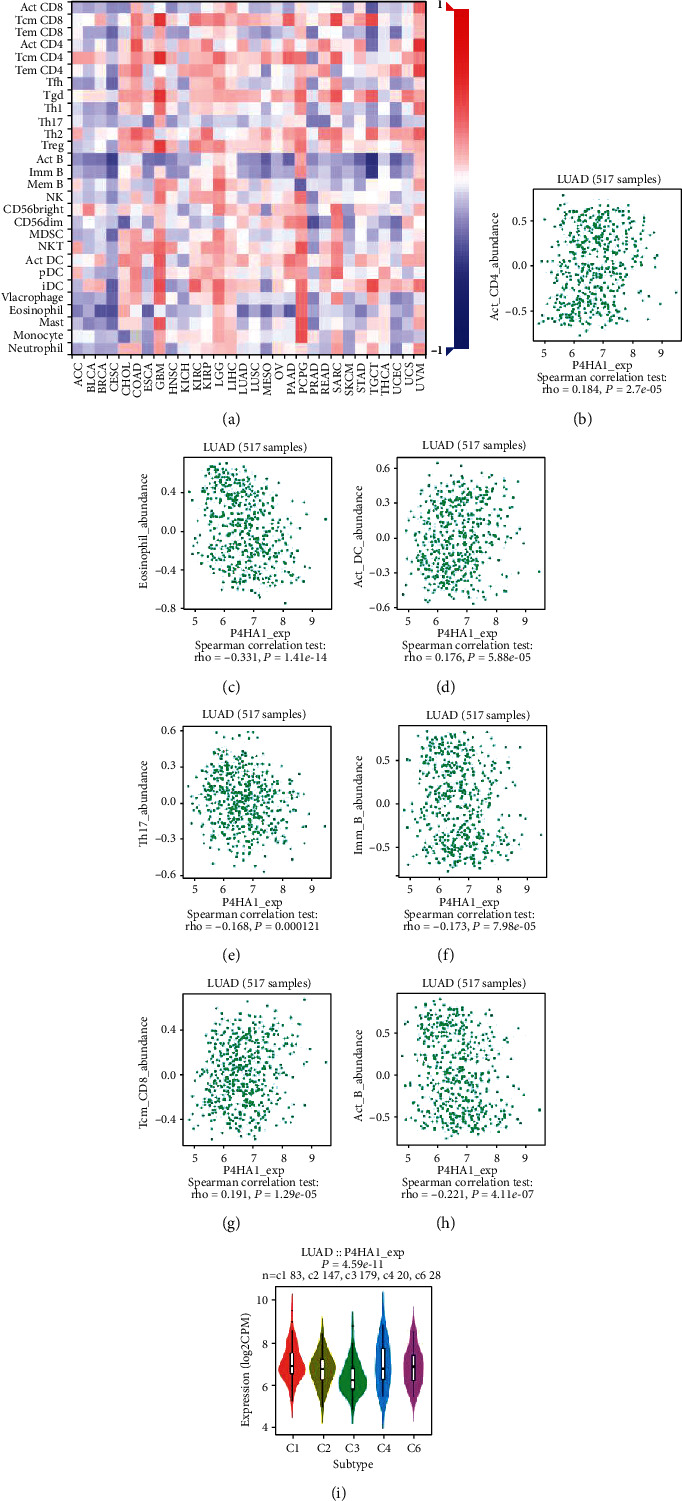
Correlation of P4HA1 expression with immune cells in LUAD based on TISIDB. (a) The landscape of relationship between P4HA1 expression and TIICs in different types of cancer (red is positive correlated and blue is negative correlated). (b–h) P4HA1 expression was positively correlated with the abundance of central memory CD8+ T cell, activated CD4+ T cell, and activated dendritic cell and was inversely related to the abundance of activated B cell, immature B cell, type 17 helper cell, and eosinophil. (i) Correlation of P4HA1 expression and immune subtypes (C1: wound healing; C2: IFN-gamma dominant; C3: inflammatory; C4: lymphocyte depleted; C5: immunologically quiet; C6: TGF-*β* dominant) in LUAD.

To investigate the potential association between P4HA1 and different immune cell infiltrations, we used the TISIDB database. The landscape of the association between P4HA1 expression and abundance of 28 TIICs in various types of cancer is shown in Figure 7(a). In LUAD, P4HA1 expression was positively correlated with the abundance of central memory CD8 T cell (rho = 0.191, *P* < 0.001), activated CD4 T cell (rho = 0.184, *P* < 0.001), and activated dendritic cell (rho = 0.176, *P* < 0.001) and was inversely related to the abundance of the activated B cell (rho = −0.221, *P* < 0.01), immature B cell (rho = −0.173, *P* < 0.001), type 17 helper cell (rho = −0.168, *P* < 0.001), and eosinophil (rho = −0.331, *P* < 0.001) (Figures 7(b)–7(h)). Then, we explore the relationship between P4HA1 expression and immune subtypes, as shown in Figure 7(i), P4HA1 expression was significantly related to immune subtypes in LUAD (*P* < 0.001).

**Figure 8:**
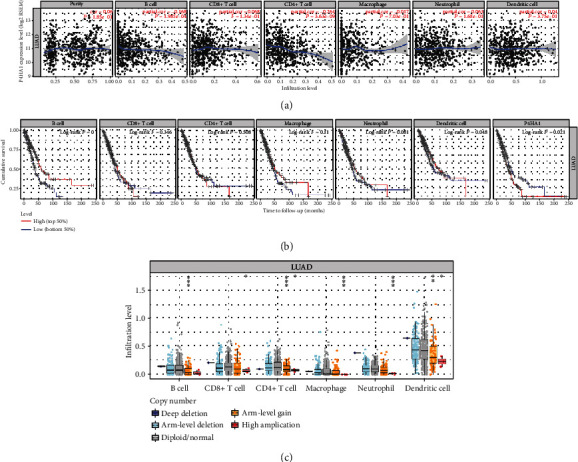
Correlation of P4HA1 expression with immune cells in LUAD based on TIMER. (a) Correlation between P4HA1 expression and abundance of immune infiltrates. (b) Clinical outcome and abundance of immune infiltrates of P4HA1 expression. (c) Correlation between somatic copy number alterations (SCNA) and abundance of immune infiltrates of P4HA1.

TIMER was further used to explore the association between P4HA1 expression and immune infiltrating levels based on the TCGA-LUAD cohort. The results showed that B cells and CD4+ T cells had a significantly negative correlation with the expression of P4HA1 in LUAD (*P* < 0.001, Figure 8(a)). Kaplan-Meier plots for TIICs and P4HA1 expression indicated that B cells and dendritic cells of immune infiltration significantly influenced the prognosis in LUAD patients (*P* < 0.05, Figure 8(b)). Boxplots for SCNAs of P4HA1 suggested that arm-level deletion and high amplification of P4HA1 were closely correlated with six infiltrating immune cells in LUAD (Figure 8(c)). However, the potential mechanism of the interplay between P4HA1 and TIICs deserves further study and exploration.

## 4. Discussion

ECM is a noncellular structure and offers physical scaffolds involving collagens, proteoglycans, fibronectin, elastin, laminins, and microfibrillar proteins for a variety of cells [25]. The collagens are major elements of ECM and play pivotal roles in maintaining various tissue structures. Meanwhile, collagens also have other plentiful, significant functions, such as cell adhesion, cell migration, tissue remodeling, and dynamic interaction between cells [26]. Previous researches reported that either increased [27] or decreased [28] deposition of collagens might be associated with increased malignancy. Collagen prolyl 4-hydroxylase (P4H) is an *α*2*β*2 tetramer and plays a vital role in all collagen deposition and biosynthesis, because the P4H residues are indispensable to fold the polypeptide chains of newly synthesized collagen into stable triple helix molecules [13, 14, 26, 29]. Moreover, P4H is essential for regulating the hypoxia-inducible factor 1*α* (HIF-1*α*) [30–32]. HIF-1 facilitates ECM remodeling through upregulating expression of P4HA1, P4HA2, and PLOD2 in hypoxic fibroblasts, which results in tumor invasion and metastasis [33]. P4HA1 is considered to be the major isoform of the P4H, which mediates three-dimensional folding of newly synthesized collagen and contributes to the majority activities of P4H [15, 34].

In our study, the P4HA1 expression at the mRNA level analyzed by the TIMER database was found to be upregulated not only in LUAD but also in various cancerous tissues. Meanwhile, upregulation of P4HA1 has been validated to facilitate carcinogenesis and progression of several cancers. In breast cancer, upregulated P4HA1 was crucial for HIF-1*α* stabilization, cancer metastasis, and chemoresistance [19, 35]. In gliomas, overexpression of P4HA1 facilitated neovascularization by transdifferentiating glioma stem cells to endothelial cells and stabilization of vascular base membranes, which contributed to tumor progression and predicted poor prognosis of gliomas [18, 36]. In prostate cancer, Chakravarthi et al. proved that overexpression of P4HA1 was essential for tumor growth and invasion in vitro and in vivo and regulated by microRNA-124 [20]. In liver cancer, Feng et al. validated that miR-30e enabled to downregulate the expression of P4HA1 at both mRNA and protein levels and inhibited the proliferation of tumor cells [37]. In pancreatic cancer, Cao et al. found that the P4HA1-HIF1*α* loop acted as a crucial regulator in glycolysis and oncogenesis and might serve as a promising therapy target [17]. A recent study showed a signature involving P4HA1, PLOD1, KDM3A, PLOD2, and ASPH predicted prognosis in various cancers including LUAD using bioinformatic analysis [38]. However, to the best of our knowledge, the expression and significance of P4HA1 in LUAD have not been investigated so far.

In the present study, we collected LUAD expression profile data from the TCGA database and GEO database and verified that P4HA1 expression was increased prominently in tumor tissues compared to normal tissues. We further investigated the association between P4HA1 expression and clinicopathologic features. We found that overexpression of P4HA1 was tightly associated with advanced clinical stage, larger primary tumor size, and shorter survival time. These results were consistent with the studies mentioned above. Univariate and multivariate Cox analyses also confirmed that elevated P4HA1, advanced stage, and distant metastasis were independent factors to predict poor OS in LUAD patients. These results showed that P4HA1 may serve as an oncogene and promote carcinogenesis and invasion in LUAD.

To explore coaltered genes along with P4HA1, we employed LinkedOmics to draw coexpression heat maps. The results were confirmed in the cBioPortal database. We found that PLOD2, ERO1L, and PGK1 were most highly coaltered along with P4HA1. Then, we constructed a PPI network based on these coaltered genes. PLOD2, ERO1L, and PGK1 were also identified as interacting partners of the P4HA1 protein. PLOD2 was reported to promote migration by inducing collagen reorganization and was regulated by the PI3K/AKT signaling pathway in NSCLC [39]. ERO1L overexpressed and contributed to a poor prognosis via modulating cell cycle-related molecules in NSCLC [40]. PGK1 was demonstrated to interact with MetaLnc9 and lead to the activation of the AKT/mTOR signaling pathway [41].

Furthermore, we explored the functions of P4HA1 in LUAD using GSEA based on TCGA data. We found the following pathways significantly enriched in the high-P4HA1 expression phenotype, such as ubiquitin-mediated proteolysis, P53 signaling pathway, cell cycle, mismatch repair, nucleotide excision repair, DNA replication, TGF-*β* signaling pathway, PI3K/AKT/mTOR signaling pathway, IL1 mediated signaling pathway, regulation of macroautophagy, targets in activated B lymphocyte, and hypoxia. All these pathways are classical cancer-related and immune-related biological processes and pathways. The ubiquitination pathway is widely involved in the regulation of cell cycle, proliferation, apoptosis, differentiation, damage repair, inflammation, immunity, and almost all other life activities [42]. Aberrant ubiquitin pathway has been functionally linked to the development and progression of many human diseases, including human tumors [43]. In lung cancer, it was reported that TRIM59 induced ABHD5 ubiquitination, leading to its proteasome-dependent degradation, whereas ABHD5 deficiency leads to metabolic reprogramming of macrophages and activation of NLRP3 inflammatory microsomes, generating an inflammatory environment for tumor development [44]. The transforming growth factor-beta (TGF-*β*) is a member of a superfamily of cytokines which plays an important role in normal development and homeostasis. A growing number of studies have demonstrated that the TGF signaling pathway plays an important role in the migration, invasion, and metastasis of lung cancer [45, 46]. DNA mismatch repair is an important genetic mechanism for maintaining DNA homeostasis in cells. Its defects will result in a phenotype called microsatellite instability (MSI), which recently received increasing attention as a significant biomarker to predict the response to cancer immunotherapy [47]. The genotype of mismatch repair genes has also been reported to be associated with the development of lung cancer. The PI3K/AKT/mTOR pathway is a signal transduction pathway that is involved in the regulation of a variety of cellular functions and is essential for the regulation of cell growth and metabolism [48]. It is closely associated with the development of non-small cell lung cancer and disease progression [49]. Inhibition of this pathway is considered a promising strategy for targeted therapy [50]. Nucleotide excision repair (NER) inhibits tumorigenesis caused by mutations of genes through repairing structurally unrelated DNA damage [51]. P53 is the most commonly mutated gene in human cancers. Mutated P53 loses its original cancer-suppressive effect. Compared with wild-type P53, mutant P53 is less sensitive to degradation, resulting in its high expression in vivo [52]. In our study, we found that the ubiquitin pathway, cell cycle pathway, TGF-*β* pathway, mismatch repair, nucleotide excision repair, PI3K/AKT/mTOR pathway, and P53 signaling pathway were upregulated in the P4HA1-high expression group. We speculate that the ubiquitin pathway, TGF-*β* pathway, and PI3K/AKT/mTOR pathway promote tumorigenesis by regulating cell cycle, interfering with apoptosis and autophagy. Tumorigenesis in turn results in increased activity of signaling pathways associated with tumor suppression, such as mismatch repair pathways and nucleotide excision repair. However, the development of tumor depends on the unbalance between promoting factors and tumor-suppressing factors. These are famous signaling pathways related to tumorigenesis, tumor development, and immune response. Therefore, it is not difficult to conclude that P4HA1 may be tightly correlated with the development and progression of LUAD. In addition, further exploration of the exact mechanism associated with P4HA1 is of enormous significance.

Compelling evidence manifested that the development and growth of tumor not only depend on the intrinsic factors of tumor cells but also on the composition of the tumor microenvironment (TME) [53–55]. The TME is a complicated network, which is rich in various infiltrating immune cells, endothelial cells, fibroblasts, and extracellular matrix [56]. The molecular features of neoplastic cells interact with TIICs dynamically in TME, which may inhibit or promote the tumor growth [57]. In recent years, with the satisfying results of immunotherapy, the exploration of infiltrating immune cells for diagnostic and prognostic value has become a hot research topic. Currently, no researchers have reported the relationship between P4HA1 and immune infiltration. Hence, we carried out immune-related analysis using TISIDB and TIMER. The results of TIMER indicated a substantial explicit connection of P4HA1 expression with infiltration levels of CD4+ T cells and B cells in LUAD. Cumulative survival curves indicated that dendritic cells and B cells had a huge impact on the prognosis of LUAD patients. The results of the TISIDB database indicated that P4HA1 expression was positively correlated with the abundance of the central memory CD8+ T cell, activated CD4+ T cell, and activated dendritic cell and was inversely related to the abundance of the activated B cell, immature B cell, type 17 helper cell, and eosinophil. Tumor-infiltrating B lymphocytes (TIBs) can be observed in all stages of lung cancer development, and abundance differs between stage and histological subtypes, which indicated that B lymphocytes exert a central role in lung cancer progression [58, 59]. TIBs were confirmed to secrete chemokines and cytokines and maintain the structure and function of the tertiary lymphoid structure in TME of cancer [60]. The presence of TIBs is extremely beneficial for long-term overall survival (OS), recurrence-free survival (RFS), or disease-specific survival (DSS) of NSCLC [61]. Therefore, P4HA1 can be served as a promising biomarker related to prognosis and immune infiltration for LUAD patients. The underlying mechanism between TIICs and P4HA1 deserves further research and exploration.

However, this study has some limitations. First, the results were not proven at the protein expression level. Second, our results were only validated in the public database and not in clinical specimens. Last, the exact mechanisms of P4HA1 involved in the development of LUAD were not clarified in this study. Therefore, prospective studies in clinical samples and mechanism researches in vivo and vitro are urgently required.

## 5. Conclusions

In general, increased P4HA1 expression has a profound impact on oncogenesis and progression of LUAD. Overexpression of P4HA1 is associated with poor prognosis. Besides, P4HA1 might participate in the development of LUAD through the following pathways, such as ubiquitin-mediated proteolysis, P53 signaling pathway, mismatch repair, nucleotide excision repair, cell cycle, and DNA replication. Meanwhile, aberrant P4HA1 expression is associated with different abundance of immune infiltrating cells (CD4+ T cells and B cells) in LUAD. Therefore, P4HA1 is a promising prognostic and immune-related biomarker for LUAD patients.

## Figures and Tables

**Table 1:** Association between P4HA1 expression and clinicopathologic characteristics using logistic regression.

Clinical characteristic	Total (*N*)	Odds ratio in P4HA1 expression	*P* value
Age (≤65 vs. >65)	494	0.78 (0.55-1.12)	0.177
Gender (female vs. male)	513	1.40 (0.98-1.99)	0.057
Stage (I vs. II/III/IV)	505	1.60 (1.28-2.29)	0.009
Tumor status (T1 vs. T2/T3/T4)	510	2.21 (1.52-3.25)	<0.001
Lymph node (N0 vs. N1/N2/N3)	501	1.52 (1.05-2.20)	0.028
Distant metastasis (M0 vs. M1)	369	2.25 (0.97-5.65)	0.066

**Table 2:** Gene sets enriched in high P4HA1 expression phenotype.

Gene set name	NES	NOM *P* val	FDR *q* val
KEGG_ubiquitin_mediated_proteolysis	2.346	0	0.002
KEGG_cell_cycle	2.096	0	0.017
KEGG_P53_signaling_pathway	1.876	0.008	0.050
KEGG_mismatch_repair	1.846	0.002	0.051
KEGG_nucleotide_excision_repair	1.967	0.010	0.035
KEGG_DNA_replication	1.769	0.015	0.059
KEGG_TGF_beta_signaling_pathway	1.828	0.018	0.051
Hallmark_PI3K_AKT_mTOR_signaling	2.079	0.002	0.007
Hallmark_hypoxia	2.195	0	0.003
GO_regulation_of_macroautophagy	2.065	0.006	0.012
GO_interleukin_1_mediated_signaling_pathway	2.161	0.008	0.008
Shaffer_IRF4_targets_in_activated_B_lymphocyte	2.263	0	0

## Data Availability

The data used for analysis in this study are available from the Cancer Genome Atlas and the Gene Expression Omnibus database freely.
